# How to Prevent or Reduce Prescribing Errors: An Evidence Brief for Policy

**DOI:** 10.3389/fphar.2019.00439

**Published:** 2019-06-12

**Authors:** Bruna Carolina de Araújo, Roberta Crevelário de Melo, Maritsa Carla de Bortoli, José Ruben de Alcântara Bonfim, Tereza Setsuko Toma

**Affiliations:** Department of Health, Institute of Health, Government of the State of São Paulo, São Paulo, Brazil

**Keywords:** inappropriate prescribing (MeSH term), prescription errors, pharmaceutical services (MeSH), evidence brief for policy, patient safety

## Abstract

- Preventing prescribing errors is critical to improving patient safety.
- We developed an evidence brief for policy to identify effective interventions to avoid or reduce prescribing errors.
- Four options were raised: promoting educational actions on prudent prescribing directed to prescribers; incorporating computerized alert systems into clinical practice; implementing the use of tools for guiding medication prescribing; and, encouraging patient care by a multidisciplinary team, with the participation of a pharmacist.
- These options can be incorporated into health systems either alone or together, and for that, it is necessary that the context be considered.
- Aiming to inform decision makers, we included considerations on the implementation of these options regarding upper-middle income countries, like the Brazilian, and we also present considerations regarding equity.

## Prescribing Errors: a Worldwide Problem

Patient safety became the focus of attention of the World Health Organization (WHO), which in 2004 launched the World Alliance for Patient Safety (World Health Organization (WHO), 2017). During the second Global Ministerial Summit on Patient Safety in 2017, the WHO Director-General announced a third challenge to be faced: drug safety.

Medication errors are a relevant problem to face, in terms of patient damage and health systems sustainability, since worldwide their costs are estimated to reach 42 billion US dollars per year. The goal proposed by WHO is to reduce the level of serious and preventable drug-related harm by 50% within a 5-year period. One of the recommendations is the development of specific action programs to improve safety in situations where a drug can cause unintended harm, including health professionals' behavior and medication practices and systems (Donaldson et al., 2017).

In this context, it is important to distinguish “medication error” and “prescribing error,” often used interchangeably in the literature. A medication error can be characterized as “a failure in the treatment process that leads to, or has the potential to lead to, harm to the patient,” which encompasses prescribing errors (Table 1), dispensing errors and administration errors (Ferner and Aronson, 2006; Ferner, 2014). Nevertheless, medication errors are difficult to assess because of the variety of terms that are misused for this purpose. Several types of errors can be influenced by different factors and result in a variety of outcomes that may require specific courses of action (Rosa et al., 2009; Ferner, 2014; Lavan et al., 2016). It is worth noting that the errors committed by prescribers are the major factor behind the occurrence of medication errors (Qureshi et al., 2011; Porter and Grills, 2016).

**Table 1:** Classification of prescribing errors.

**Prescribing errors**	
Omission error	Suppression of a drug previously used
Commission error	Addition of a drug not previously used
Dosing error	Incorrect dose
Frequency error	Incorrect dose frequency
Pharmaceutical form error	Incorrect pharmaceutical form
Substitution error	A drug from one class is substituted for another drug from the same class not previously used
Duplication error	Two drugs from the same class are prescribed

*Adapted from Lavan et al. (2016)*.

Prescribed drugs are considered to rank as the third leading cause of death in the United States and Europe, surpassed only by heart disease and cancer. While about 100,000 deaths each year in the United States could be related to people taking drugs correctly, another equivalent number of deaths would occur due to errors like the use of contraindicated drugs or in very large doses. Impotent drug regulation, corruption of scientific evidence, drug marketing, and bribery of physicians are pointed out as factors that contribute to this situation (Gøtzsche, 2014).

In India, drug misuse is also common, and the major determinants of the problem include the lack of effective regulation and education on the appropriate use of these products. It is estimated that ~50% of the average family spending on medicines is unreasonable or unnecessary (Porter and Grills, 2016).

In Brazil, Martins et al. (2011) analyzed medical records of 103 patients from three different hospitals and found that the occurrence of avoidable adverse events was 2.3%, whereas the mortality rate related to adverse events was about 8.5%. Among the elderly individuals, a use prevalence of 11.5–62.5% of potentially inappropriate drugs was associated with adverse effects, hospitalization, morbidity, mortality, and a higher cost of health services (Lucchetti and Lucchetti, 2017).

In this context, this study was aimed at identifying evidence in the scientific literature of effective interventions to avoid or reduce prescribing errors.

## Support Tools for Drawing up Evidence Briefs for Policy

This is an evidence brief for policy that followed the methodological guidelines proposed by the SUPPORT collaboration group—Supporting Policy Relevant Reviews and Trials (Lavis et al., 2009).

Evidence briefs for policy are documents that identify, through the most reliable scientific evidences, interventions to deal with a policy-related issue. They are tailored to inform decision makers on the best available and efficient actions to handle with health policy problems, without posing a recommendation, since the process of decision making depends on a variety of factors, including the local context. Within this structure, it is usually found a problem and its relevance for health policies, options to deal with the problem, considerations regarding implementation and equity (Bortoli et al., 2017).

The search for studies was carried out in December 2017, in nine databases: BVS Regional Portal; PubMed, Health Systems Evidence; Health Evidence, PDQ-Evidence; Center for Reviews and Dissemination; Embase; Cochrane Database of Systematic Reviews; and Epistemonikos. In our search strategy, we used the terms “Inappropriate Prescribing” and “Prescription Errors.” Search filters were used for identifying systematic reviews published in English, Spanish, and Portuguese. This process was performed by a researcher from our team, and no limits were placed on the publication date.

Article selection and data extraction were carried out independently by two investigators, and disagreements were resolved by a third investigator. The studies thus identified that did not fit our inclusion criteria (systematic reviews, strategies/interventions to enhance prescribing, strategies that involved not only physicians) were excluded after reading their titles, abstracts and full texts (Supplementary Table S1). Data from the selected systematic reviews were extracted into a spreadsheet containing information related to the study population, interventions administered, outcomes, and countries according to their income (Supplementary Table S2). From this extraction, we came up with a range of interventions, which were arranged in groups according to their similarity, resulting in options for dealing with the problem.

The methodological quality of the selected systematic reviews was assessed independently by two investigators who used the Assessing Methodological Quality of Systematic Reviews tool—AMSTAR (Shea et al., 2007). Any divergences were settled by consensus.

In order to implement health policies, it is necessary to reflect on their implications so as not to cause or increase health iniquities. In this study, we used the tool PROGRESS—an acronym standing for Place of residence; Race/ethnicity/culture/language; Occupation; Gender/sex; Religion; Education; Socioeconomics status; Social capital (Evans and Brown, 2003)—for making considerations on equity in the policy options.

Most systematic reviews included in the options were developed in HIC (high-income countries), thus, in order to best address considerations about the process of implementation, for each one of the options we searched qualitative articles at the BVS Regional Portal. This step aimed to identify, preferably, strategies performed in Brazil, our context, and that could be relatable to other UMIC (Upper-Middle-Income Countries).

## Policy Options for Preventing or Reducing Prescribing Errors

**Figure 1:**
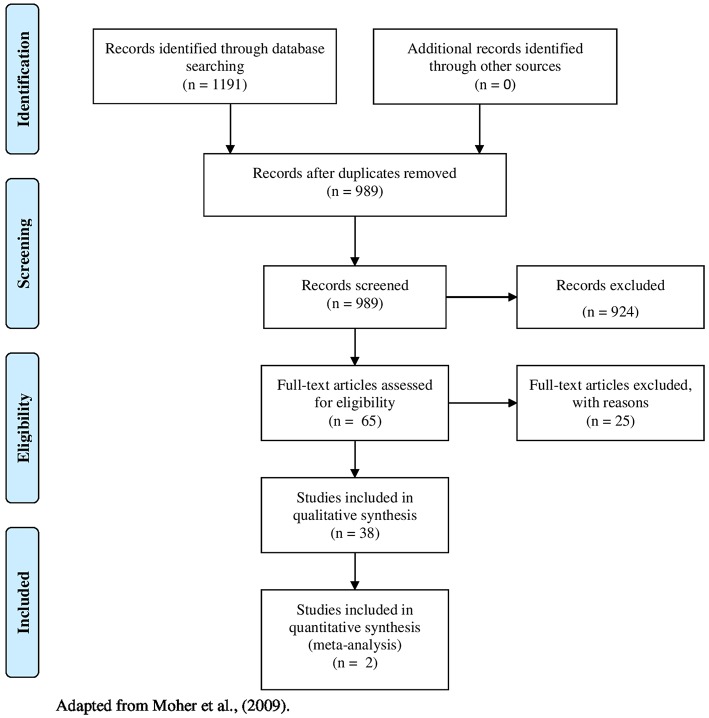
Flow diagram for study selection. Adapted from Moher et al. (2009).

Of the 1,191 systematic reviews identified, 40 were selected and analyzed in order to draw up the options provided (Figure 1). From the set of interventions extracted from the systematic reviews, we devised four options for dealing with prescribing errors, which we present below: (1) Promoting educational actions on prudent prescribing directed to prescribers; (2) Incorporating computerized alert systems into clinical practice; (3) Implementing the use of tools for guiding medication prescribing; and (4) Encouraging patient care by a multidisciplinary team, with the participation of a pharmacist.

### Option 1. Promoting Educational Actions on Prudent Prescribing Directed to Prescribers

Thirteen systematic reviews addressed the effectiveness of educational actions in preventing or reducing prescribing errors, of which six were deemed as having high methodological quality, three as moderate, and four of low quality.

The following studies highlighted the positive effects produced by educational actions through different approaches: educational performance of pharmacists (Ross and Loke, 2009; Tesfaye et al., 2017); actions that improve the transfer of information among prescribers and discussion of cases in the multidisciplinary team in long–term care facilities for the elderly (Alldred et al., 2016); educational actions with multidisciplinary teams (Chiatti et al., 2012); distribution of clinical protocols and therapeutic guidelines, educational meetings, audit and feedback (Arnold and Straus, 2005); small group workshops, use of decision trees, sharing of quarterly reports, and annual educational actions (Kaur et al., 2009); educational initiatives dissemination, targeted studies and meetings with the participation of professionals (Forsetlund et al., 2011); use of web-based education program, use of performance feedback, along with patient and clinician education, direct and individualized education actions (Brennan and Mattick, 2013); multifaceted interventions (Brennan and Mattick, 2013; Ivanovska and Holloway, 2013; Roque et al., 2014; Coxeter et al., 2015); educational actions that promote behavior change (Tonkin-Crine et al., 2011) tailored to antimicrobial stewardship teams (Davey et al., 2017); interactive educational workshops with reinforcement by a local opinion leader (Fleming et al., 2013).

All reviews concluded that different educational interventions can be effective in reducing inappropriate prescriptions.

### Option 2. Incorporating Computerized Alert Systems Into Clinical Practice

Eighteen systematic reviews, of which eleven were classified as high methodological quality, three of moderate one and four as low quality, addressed the use of electronic systems and showed the effectiveness of using different systems in reducing prescribing errors.

The studies emphasized a positive effect on improving prescription writing or reducing prescribing errors by using: alert systems (Schedlbauer et al., 2009; Davey et al., 2017); drug dose adjustment supported by information technology (Mekonnen et al., 2016); electronic archives in hospitals (Sánchez et al., 2014); electronic prescribing resources for undergraduate students (Ross and Loke, 2009); medical reminders, information provided at the time of prescription writing on an online prescription editor (Arnold and Straus, 2005); a Clinical Decision Support System (Kaushal et al., 2003; Yourman et al., 2008; Kaur et al., 2009; Pearson et al., 2009; Reckmann et al., 2009; Lainer et al., 2013; Maaskant et al., 2015; Clyne et al., 2016); a Medical Order Entry System (Kaur et al., 2009) at an intensive care unit (Kaushal et al., 2003; Hodgkinson et al., 2006; Van Rosse et al., 2009; Khajouei and Jaspers, 2010); a Prescription Automatic Screening System (Yang et al., 2012).

Nevertheless, some studies have shown increased medication and prescribing errors when using complex Physician Order Entry Systems (Khajouei and Jaspers, 2010), due to excessive available information (Lainer et al., 2013).

### Option 3. Implementing the Use of Tools for Guiding Medication Prescribing

Nine systematic reviews, four of which were considered to be of high methodological quality, four of moderate and three of low quality, provided information on the use of medication prescribing tools.

The findings showed that the tools that may be useful for improving prescribing quality and reducing inadequate prescription are: STOPP/START (Cooper et al., 2015; Santos et al., 2015; Hill-Taylor et al., 2016; Hyttinen et al., 2016) and Beers criteria (Garcia, 2006; Jano and Aparasu, 2007; Soares et al., 2011; Cooper et al., 2015; Santos et al., 2015; Hyttinen et al., 2016). In addition, these tools can be combined with other actions, such as educational ones (Alldred et al., 2016; Valencia et al., 2016).

STOPP - Screening Tool of Older Persons' Prescriptions and START - Screening Tool to Alert to Right Treatment are prescribing screening tools for older people (Mahony et al., 2010).

Beers criteria are lists of potentially inappropriate drugs for the elderly (DeSevo and Klootwyk, 2012).

### Option 4. Encouraging Patient Care by a Multidisciplinary Team, With the Participation of a Pharmacist

Nine systematic reviews, of which four were regarded as being of high methodological quality, three of moderate and three of low quality, showed that working as a multidisciplinary team reduces prescribing errors, especially when there is a pharmacist in the team (Chiatti et al., 2012; Sánchez et al., 2014; Alldred et al., 2016; Clyne et al., 2016).

These studies indicated that, as far as patient care is concerned, a multidisciplinary team is better indicated to reduce inappropriate or multiple prescribing (Garcia, 2006; Kaur et al., 2009), decrease inappropriate prescribing in elderly patients (Riordan et al., 2016; Walsh et al., 2016), and antibiotic inappropriate prescribing (Fleming et al., 2013;Maaskant et al., 2015).

## Considerations About Implementing Policy Options and Their Equity

Although the options presented, do not necessarily have to be implemented together nor in a comprehensive way, their practical implementation should consider local feasibility and whether they can be integrated into the governability of decision making, irrespective of a health system's size (whether national, regional, or local). When implementing health policy options, managers usually need to tackle several types of obstacles. Not only it is necessary to consider them, but also to find ways to overcome them, especially those related to cultural and social representations of health care users and workers. The following are some difficulties that may be encountered when implementing each of the options and issues that may give rise to iniquities, especially in Upper-Middle Income Countries.

### Option 1. Promoting Educational Actions on Prudent Prescribing Directed to Prescribers

Implementing these interventions may aggravate iniquities when the prescriber does not participate in those activities, whatever the reasons, which may be a consequence of institutional disorganization, lack of personal motivation, or overvaluation of the knowledge they already have.

In the literature, the barriers that must be overcome may be encountered both at the individual level (courses and training of their interest and a belief that empirical knowledge is enough on its own), and at the collective level (communication difficulties among teams, infrastructure, a lack of available time to perform those activities, and punitive management, all of which can have a negative impact on professionals). In addition, difficulties may arise due to insufficient human resources or in complying with previously established guidelines (Carvalho et al., 2011; Bonadiman et al., 2013; Marchon and Mendes, 2014; Ugarte and Acioly, 2014; Santos, 2016; Silva, 2016).

### Option 2. Incorporating Computerized Alert Systems Into Clinical Practice

It should be highlighted that the implementation of these electronic resources requests some infrastructure (for example, computer or Internet access, human resources for support), as well as actions to raise awareness about and encourage the use of these technologies by prescribers.

The obstacles observed include a lack of rapid and simplified access to information by means of electronic systems in emergency situations (Cassiani et al., 2003; Gimenes et al., 2006), a lack of culture regarding the adequate inputting of information into the system (Cassiani et al., 2003; Marchon and Mendes, 2014), and a lack of participation in trainings aimed at enhancing the understanding of how the electronic system actually works. It is also important to note that these systems require financial resources, which can make them difficult to deploy (Freire et al., 2004).

### Option 3. Implementing the Use of Tools for Guiding Medication Prescribing

These tools are tailored for use mostly in the elderly population, which therefore limits their use in the entire population. Furthermore, the difficulty of access or even the lack of knowledge about these resources precludes them from being used in the clinical practice (Jano and Aparasu, 2007; Soares et al., 2011; Hill-Taylor et al., 2016; Hyttinen et al., 2016; Valencia et al., 2016).

Based on the tools, it can be noted that the lack of knowledge about the resources (Miasso et al., 2006), not considering specific characteristics of the patient (Hyttinen et al., 2016) and the constant updates (Soares et al., 2011) are all obstacles to their incorporation and use.

### Option 4. Encouraging Patient Care by a Multidisciplinary Team, With the Participation of a Pharmacist

Among the barriers that we found, there are a reduced number of professionals, work overload, a lack of communication among team members (Silva et al., 2007), not to mention resistance to incorporating the pharmacist into the care management staff.

In addition, we have also observed that verbal interaction among professionals (pharmacists and doctors) alone, does not produce significant results (Silva, 2016). Not sharing the patients' clinical data (medical records, for example) with all professionals that exert an influence over the therapeutic conduct, hamper prescription validation (Cardinal and Fernandes, 2014). It should also be emphasized that inadequate resources may prevent professionals from being employed or replaced.

## Evidence Gaps

Further studies should be conducted on factors influencing prescribing and evaluating specific strategies (Davey et al., 2017). High-quality studies assessing the effectiveness of educational actions are still scarce in the literature (Alldred et al., 2016).

Pearson et al. (2009) reported that further studies should analyze the benefits of automated prescribing screening systems, since there is a lack of studies on the impact of the system on drug-related adverse events, safety, quality, cost, and patient outcomes (Yang et al., 2012). Evidence of effective interventions based on computerized systems to prevent medication errors in the pediatric inpatient population is also incipient (Maaskant et al., 2015). Further research is also needed to check the effectiveness of the strategies found in the implementation of computerized alert tools (Kaushal et al., 2003; Hodgkinson et al., 2006), as well as to assess the impact of interventions on legibility and completeness of electronic prescriptions (Reckmann et al., 2009).

The use of the STOPP/START criteria remains incipient in health services, except in emergency services, and further studies are thus needed to assess this tool's efficacy in detecting potentially inappropriate prescriptions (Hill-Taylor et al., 2016).

## Conclusion

There are several options indicated in the scientific literature that are effective and safe to assist professionals in order to avoid or reduce medication prescribing errors in health services. Our evidence brief for policy present four options that may be useful to deal with this problem, although there is no recommendation on which one is the best. The decision to implement one or more options depends on the context where the decision makers are inserted.

The options are not exclusive and can be used together, according to the local reality of implementation.

When implementing these options, however, it should be taken into account that the number of studies is still incipient and confidence in the results could be improved with further research with high methodological quality.

## Author Contributions

BA, RM, JB, and TT contributed with the design and conception of the study. BA and RM wrote the first draft of the manuscript. BA, RM, and TT participated in the study selection process. MB and TT contributed to the revision of the manuscript, read and approved the submitted version.

### Conflict of Interest Statement

The authors declare that the research was conducted in the absence of any commercial or financial relationships that could be construed as a potential conflict of interest.
