# Supplementary material for: How to Prevent or Reduce Prescribing Errors: An Evidence Brief for Policy
Source: Front Pharmacol. 2019 Jun 12;10:439. doi: 10.3389/fphar.2019.00439 (PMC6584796; doi:10.3389/fphar.2019.00439)
Supplement: Supplementary file 1 [file Table_1.DOCX]

**Supplementary table S1 – characteristics of excluded studies.**

| **Author/year** | **Title** | **Reason for Exclusion** |
| --- | --- | --- |
| AL SHEMELI; STEWART, 2014 | Use of the Drug Burden Index to identify and reduce potentially inappropriate prescribing of anticholinergic and sedative agents in elderly patients in institutionalized care: A systematic review protocol. | No access to complete information |
| BOONACKER et al., 2010 | Interventions in health care professionals to improve treatment in children with upper respiratory tract infections | Not a strategy to prevent or reduce prescribing errors |
| BOS et al., 2017 | The effect of prescriber education on medication related patient harm in the hospital: A systematic review | Does not include a strategy |
| CHARANI et al., 2011 | Behavior change strategies to influence antimicrobial prescribing in acute care: a systematic review. | Does not include a strategy |
| CLYNE, B. et al., 2012 | Electronic prescribing and other forms of technology to reduce inappropriate medication use and polypharmacy in older people: a review of current evidence. | Not a systematic review |
| CORSONELLO et al., 2012 | Explicit criteria for potentially inappropriate medications to reduce the risk of adverse drug reactions in elderly people: From beers to STOPP/START criteria. | Not a systematic review |
| COX; JONES, 2001 | Is it possible to decrease antibiotic prescribing in primary care? An analysis of outcomes in the management of patients with sore throats. | Not a systematic review |
| CULLINAN et al., 2014 | A meta-synthesis of potentially inappropriate prescribing in older patients. | Does not include a strategy |
| DI GIORGIO; PROVENZANI; POLIDORI, 2016 | Potentially inappropriate drug prescribing in elderly hospitalized patients: an analysis and comparison of explicit criteria. | Not a systematic review |
| FORTUNA et al., 2008 | Clinician attitudes towards prescribing and implications for interventions in a multi-specialty group practice. | Not a systematic review |
| HO; VENCI, 2012 | Improving the success of mailed letter intervention programs to influence prescribing behaviors: a review. | Not a systematic review |
| KOJIMA et al., 2016 | Screening tool for older persons. Appropriate prescriptions Japanese: Report of the Japan Geriatrics Society Working Group on “Guidelines for medical treatment and its safety in the elderly. | Not a systematic review - Guideline development |
| LOPEZ et al., 2012 | Interventions for reducing medication errors in hospitalised adults. | Systematic review protocol |
| LUCAS et al., 2015 | A systematic review of parent and clinician views and perceptions that influence prescribing decisions in relation to acute childhood infections in primary care. | Does not include a strategy |
| OREN; SHAFFER; GUGLIELMO, 2003 | Impact of emerging technologies on medication errors and adverse drug events. | Not a systematic review |
| PAGE et al., 2016 | The feasibility and effect of deprescribing in older adults on mortality and health: a systematic review and meta-analysis. | Not a strategy to prevent or reduce prescribing errors |
| POUDEL et al., 2014 | A systematic review of prescribing criteria to evaluate appropriateness of medications in frail older people. | Does not include a strategy |
| ROSS et al., 2009 | What is the scale of prescribing errors committed by junior doctors? A systematic review. | Does not include a strategy |
| SALMI et al., 2015 | Physicians’ knowledge, perceptions, and behaviour towards antibiotic prescribing: A systematic review of the literature. | Does not include a strategy |
| SMITH et al., 2013 | Classification and definition of misuse, abuse, and related events in clinical trials: ACTTION systematic review and recommendations. | Does not include a strategy |
| Stewart et al., 2016 | Guidance to manage inappropriate polypharmacy in older people: systematic review and future developments. | Does not include a strategy |
| TAM et al., 2005 | Frequency, type and clinical importance of medication history errors at admission to hospital: a systematic review. | Not a strategy to prevent or reduce prescribing errors |
| THOMPSON et al., 2009 | Second opinions improve ADHD prescribing in a medicaid-insured community population. | Not a systematic review |
| TOPINKOVÁ et al., 2012 | Evidence-based strategies for the optimization of pharmacotherapy in older people. | Not a strategy to prevent or reduce prescribing errors |
| WATKINS et al. 2004 | Factors affecting feasibility and acceptability of a practice-based educational intervention to support evidence-based prescribing: a qualitative study. | Not a systematic review |
