# Supplementary material for: How to Prevent or Reduce Prescribing Errors: An Evidence Brief for Policy
Source: Front Pharmacol. 2019 Jun 12;10:439. doi: 10.3389/fphar.2019.00439 (PMC6584796; doi:10.3389/fphar.2019.00439)
Supplement: Supplementary file 2 [file Table_2.DOCX]

Supplementary table S2. Characteristics of included systematic reviews for raising health policy options

| **Author, year** | **Policy options** | **Strategy to reduce or prevent prescribing errors** | **Number of primary studies and participants** | **Countries** | **Intervention lenght** | **Bias in primary studies** | **AMSTAR** |
| --- | --- | --- | --- | --- | --- | --- | --- |
| Alldred et al., 2016. | 1, 3 and 4 | Educational actions for prescribers  Criteria for Beers, MAI and STOP/START  Multidisciplinary Team | 12 studies:  Design not reported  10,953 eldery individuals | HIC: 10 | 6 months -24 months | Heterogeneous studies, with high risk of bias in the blinding | 11/11 |
| Arnold; Straus, 2005 | 1 and 2 | Printed educational materials for physicians, auditing and feedback, educational meetings, educational visits, and multifaceted interventions  Computerized reminders and electronic support for proper prescription | 39 studies:  25 RCT  1 QRCT  11 CBA  2 ITS  10.250 participants | LMC: 4  UMC: 1  HIC: 12 | Not reported | Not reported | 9/11 |
| Brennan; Mattick, 2013 | 1 | Distribution of educational materials, audio-visual materials and electronic publications, meetings and training of prescribers, auditing and feedback | 64 studies:  46 pre-test and post-test studies  6 RCT  7 no-RCT  5 transversal studies  Participants not reported | LMC: 2  UMC: 4  HIC: 14 | Not reported | Risk of bias fromobserver | 6/11 |
| Chiatti et al., 2012 | 1 and 4 | Education programs.  Development of multidisciplinary team | 21 studies:  Design not reported  Participants not reported | HIC: 9 | Not reported | Not reported | 4/11 |
| Clyne et al., 2016 | 2 and 4 | Clinical Decision Support Systems (CDSS)  Inclusion of pharmacist as a team member | 12 studies:  11 RCT  1 congress study  202 participants | HIC: 7 | 6 months – 24 months | Heterogeneous studies, eith risk of bias in randomization, allocation and blinding | 9/11 |
| Cooper et al., 2015 | 3 | Criteria of Beers, STOPP, MAI, AUM and ACOVE | 12 studies:  8 RCT  2 cluster-RCT  2 controlled before and after  2,169 participants | HIC: 5 | 3 months– 12 months | Potential risk of bias | 10/11 |
| Coxeter et al., 2015 | 1 | Training in education and communication, interactive workshops, seminary, web-based platforms, use of videos, interactive exercises and interactive books | 10 studies:  Design not reported  Participants not reported | HIC: 8 | 3 months – 41 months | Moderate to low risk of bias | 11/11 |
| Davey et al., 2017 | 1 and 2 | Audit and feedback; interactive workshops led by physicians or facilitators (pharmacists) and seminary  Computerized Reminders | 210 studies:  58 RCT  138 ITS  6 controlled before-after studies  8 no -RCT  Participants not reported | LMC: 2  UMC: 6  HIC: 25 | 6 months – 36 months | 56 studies at high risk of bias and 165 at low risk | 11/11 |
| Fleming;  Browne; Byrne, 2013 | 1 and 4 | Small workshop group  Multidisciplinary groups | 4 studies:  Design not reported  11,271 physicians | HIC: 3 | 3-12 months | 2 at high risk of bias and 2 at lowrisk | 6/11 |
| Forsetlund et al., 2011 | 1 | Educational outreach initiatives, isolated educational meetings or as part of a complex intervention, audits, and peer feedback programs | 20 studies:  20 RCT  339 health professionals | HIC: 5 | 3-12 meses | 5 studies at high risk of bias and 14 at moderate risk | 9/11 |
| Garcia, 2006 | 3 and 4 | Criteria of Beers  Obtain recommendations from pharmacists to reduce inappropriate prescribing and adverse drug events | 19 studies:  Design not reported  Participants not reported | Not reported | Not reported | Not reported | 1/11 |
| Hill-Taylor et al., 2016 | 3 | Criteria STOPP/START | 4 studies:  4 RCT  1925 participants | HIC: 4 | 1 – 2 years | 2 at moderate to high risk of bias and 2 at low risk | 10/11 |
| Hodgkinson et al., 2006 | 2 | Computerized Provider Order Entry (CPOE) and Computerised Clinical Decision Support Systems (CDSS) | 20 studies:  2 systematic reviews  4 RCT  2 controlled studies  10 before-after studies  2 time series interrupted  Participants not reported | HIC: 4 | 12 weeks – 4 years | Not reported | 10/11 |
| Hyttinen; Jyrkkä; Valtonen, 2016. | 3 | Beers Criteria, STOPP/START and MAI | 39 studies:  21 retrospective cohort  7 prospective cohort  3 retrospective and prospective  4 case-control  4 RCT  1.807.404 elderly individuals | HIC: 11 | 2 months- 12 years | Low risk of bias | 7/11 |
| Ivanovska; Holloway, 2013 | 1 | Education of prescribers, printed materials, auditing and / or commentary, review of drug use, essential medicines program (list of essential medicines) | 8 studies:  7 pre-post-measurements with a comparison group.  1 RCT  1.141.649 participants | UMC: 5  HIC: 1 | Not reported | Not reported | 3/11 |
| Jano; Aparasu, 2007 | 3 | Beers Criteria | 12 studies:  8 retrospective cohort  2 prospective cohort  2 retrospective cross-sectional  557.823 participants | UMC: 1  HIC: 5 | 3 months –  7 years | Not reported | 3/11 |
| Kajouei; Jaspers, 2010 | 2 | Computerized Provider Order Entry (CPOE) | 19 studies:  Design not reported  Participants not reported | Not reported | Not reported | Not reported | 3/11 |
| Kaur et al, 2009 | 1 and 2 | Small workshop group, use of decision tree, annual visits and meetings, continuing education to physicians in conjunction with inappropriate drug tools  Computerised Clinical Decision Support Systems (CDSS) | 24 studies:  Design not reported  124. 802 participants | Not reported | Up to 1 year | Not reported | 3/11 |
| Kaushal; Shojania; Bates, 2003 | 2 | Computerized Provider Order Entry (CPOE) and Computerised Clinical Decision Support Systems (CDSS) | 12 studies:  6 RCT  2 RCT crossover  1 prospective before-after  1 retrospective  before-after  1 retrospective  time series  1 study design  2.050 participants | Not reported | Not reported | Not reported | 3/11 |
| Lainer; Mann; Sönnichsen, 2013 | 2 | Computerised Clinical Decision Support Systems (CDSS) with Computerized Provider Order Entry (CPOE), and  pharmacist led and telemedicine studies | 10 studies:  10 RCT  71.499 participants | HIC: 3 | 3 - 30 months | Moderate risk of bias | 9/11 |
| Maaskant et al., 2015 | 2 and 4 | Computerized Provider Order Entry (CPOE)  Participation of a clinical pharmacist in a clinical team | 7 studies:  2 RCT  2 interrupted time series  3 controlled before-after studies  44.132 participants | HIC: 4 | 1 - 9 months | Low risk of bias | 10/11 |
| Mekonnen et al., 2016 | 2 | Electronic medication reconciliation intervention | 10 studies:  Design not reported  21.486 pacients | HIC: 2 | 10 – 70 weeks | 5at high risk of bias and 5 at moderate risk | 11/11 |
| Pearson et al., 2009 | 2 | Computerised Clinical Decision Support Systems (CDSS) | 56 studies:  50 RCT  6 Q-RCT  Participants not reported | Not reported | Not reported | High and moderate risk of bias | 10/11 |
| Reckmann et al., 2009 | 2 | Computerized Provider Order Entry (CPOE) | 12 studies:  7 pre-post studies  2 time series  1 cross-sectional  1 crossover  1 comparative cohort  883 participants | HIC: 3 | 4 months - 14 months | Not reported | 5/11 |
| Riordan et al., 2016 | 4 | Pharmacist-led interventions | 5 studies:  4 RCT  1 interrupted temporal series  Participants not reported | HIC: 3 | Not reported | 3 studies at high risk of bias and 1 unclear | 10/11 |
| Roque et al., 2014 | 1 | Dissemination of self-explanatory educational materials, bulletins on drugs; group education and workshops | 78 studies:;  Design not reported  853.176 participants | UMC: 4  HIC: 9 | 8 weeks - 54 months | Not reported | 4/11 |
| Ross; Loke, 2009 | 1 and 2 | Introduction of a good prescription course based on the World Health Organization (WHO) Good Prescribing Guide for medical students  Electronic Prescription Resources for Undergraduate Students | 22 studies:  Design not reported  485 medical estudents | LIC: 1  HIC: 7  Unidentified countries: 10 countries | Not reported | Risk of selection bias | 9/11 |
| Sánchez; Bravo; Morales, 2014 | 2 and 4 | Electronic Prescription System  Team with the participation of the pharmacist | 8 studies:  8 prospective studies  94.251 participants | LMC: 1  UMC: 2  HIC: 4 | 1 month - 4 years | Not reported | 1/11 |
| Santos et al., 2015 | 3 | Beers Criteria and STOPP | 119 studies:  Design not reported  Participants not reported | Not reported | Not reported | Not reported | 4/11 |
| Schedlbauer et al., 2009 | 2 | Computerized Alerts and  Prompts | 20 studies:  Design not reported  Participants not reported | HIC: 3 | Not reported | Not reported | 4/11 |
| Soares et al., 2011 | 3 | Beers Criteria | 10 studies:  5 prevalence studies  2 comparison studies  1 incidence study  1 frequency study  1 evaluation study  1.231.837 participants | UMC: 1  HIC: 5  Unidentified: 8 european countries | Not reported | Not reported | 4/11 |
| Tesfaye et al., 2017 | 1 | Educational intervention of physicians, feedback from pharmacists to physicians, training followed by programs (softwares) | 49 studies:  Design not reported  146.612 participants | LIC: 2  LMC: 2  UMC: 3  HIC: 13 | Not reported | Low and moderate risk of bias | 8/11 |
| Tonkin-Crine , Yardley, Little, 2011 | 1 | Strategies that allow clinicians to reflect on their prescription | 12 studies:  12 Non-randomised  controlled trial  321 participants | HIC: 10 | Not reported | Not reported | 2/11 |
| Valencia et al., 2016 | 3 | Beers Criteria, STOPP/START, ACOVE, MAI, and AOU | 19 studies:  12 RCT  5 Non-randomised  controlled trial  2 controlled trials  Participants not reported | HIC: 8 | 1-12 months | High risk of bias | 4/11 |
| Van Rosse et al., 2009 | 2 | Computerized Physician Order Entry (CPOE) | 11 studies:  8 retrospective cohort  3 prospective cohort  1 controlled cross- sectional trial  Participants not reported | Not described | Not reported | Not reported | 4/11 |
| Walsh et al., 2016 | 4 | Pharmacists’ interventions | 8 studies:  4 studies  2 RCT  2 Non-randomised  controlled trial  1.164 patients | HIC: 3 | Not reported | Moderate risk of bias | 10/11 |
| Yang et al., 2012 | 2 | Prescription Automatic Screening System (PASS) | 12 studies:  12 before-after studies  Participants not reported | UMC: 1 | Not reported | Risk of selection bias | 10/11 |
| Yourman; Concato; Agostini, 2008 | 2 | Computer Decision Support (CDS) | 10 studies:  5 RCT  3 Cohort  2 Interrupted time series  176.030 participantes | HIC: 3 | Not reported | Not reported | 5/11 |

Legend: High income (HIC); Low income (LIC); Lower middle income (LMC); Upper middle income (UMC), World Bank list of economies (June 2018 - https://datahelpdesk.worldbank.org/knowledgebase/articles/906519-world-bank-country-and-lending-groups).
